# Predicting Spinal Cord Injury Prognosis Using Machine Learning: Systematic Review and Meta-Analysis

**DOI:** 10.2196/66233

**Published:** 2025-12-05

**Authors:** Linxing Zhong, Qiying Huang, Hao Zhang, Liang Xue, Yehuang Chen, Jianwu Wu, Liangfeng Wei

**Affiliations:** 1 Department of Neurosurgery, Fuzong Clinical Medical College of Fujian Medical University, 156 West Second Ring North Road, Fuzhou, 350025, China, 86 13960760177, 86 591-87640785

**Keywords:** artificial intelligence in health care, spinal cord injury, predictive analytics, clinical outcomes research, PRISMA, Preferred Reporting Items for Systematic Reviews and Meta-Analyses

## Abstract

**Background:**

Spinal cord injury (SCI) is complicated and varied conditions that receive a lot of attention. However, the prognosis of patients with SCI is increasingly being predicted using machine learning (ML) techniques.

**Objective:**

This study aims to evaluate the efficacy and caliber of ML models in forecasting the consequences of SCI.

**Methods:**

Literature searches were conducted in PubMed, Web of Science, Embase, PROSPERO, Scopus, Cochrane Library, China National Knowledge Infrastructure, China Biomedical Literature Service System, and Wanfang databases. Meta-analysis of the area under the receiver operating characteristic curve of ML models was performed to comprehensively evaluate their performance.

**Results:**

A total of 1254 articles were retrieved, and 13 eligible studies were included. Predictive outcomes included spinal cord function prognosis, postoperative complications, independent living ability, and walking ability. For spinal cord function prognosis, the area under the curve (AUC) of the random forest algorithm was 0.832, the AUC of the logistic regression algorithm was 0.813 (95% CI 0.805-0.883), the AUC of the decision tree algorithm was 0.747 (95% CI 0.677-0.802), and the AUC of the XGBoost (extreme gradient boosting) algorithm was 0.867. For postoperative complications, the AUC of the random forest algorithm was 0.627 (95% CI 0.441-0.812), the AUC of the logistic regression algorithm was 0.747 (95% CI 0.597-0.896), and the AUC of the decision tree algorithm was 0.688. For independent living ability, the AUC of the classification and regression tree model was 0.813. For walking ability, the model based on the vector machine algorithm was the most effective, with an AUC of 0.780.

**Conclusions:**

The ML models predict SCI outcomes with relative accuracy, particularly in spinal cord function prognosis. They are expected to become important tools for clinicians in assessing the prognosis of patients with SCI, with the XGBoost algorithm showing the best performance. Prediction models should continue to advance as large data are used and ML algorithms develop.

## Introduction

Spinal cord injury (SCI) is one of the most devastating diseases. From 1990 to 2019, the global prevalence of SCI increased by 81.5% (74.2%‐87.1%) and the incidence increased by 52.7% (30.3%‐69.8%), with an annual rise [1]. The injury has severe impacts on patients, potentially leading to varying degrees of motor, sensory, and autonomic dysfunction [2]. Furthermore, SCI imposes a heavy burden on the health care system and socio-economics. Statistics show that the average lifetime rehabilitation cost for patients with SCI may exceed US $750,000, resulting in an estimated annual expenditure of US $6 billion for SCI in the United States [3]. Since there are still no effective treatments for SCI, the actual cost of treatment keeps going up every year. Due to the complexity of SCI, it has become a global public health issue, with significant public concern regarding its treatment, rehabilitation, and prognosis. The prognosis of SCI is especially important for clinical doctors. Early assessment of patients with SCI is crucial to prevent overtreatment and to provide early personalized treatment for those with favorable prognostic opportunities, thus improving patient outcomes and facilitating their return to home and society as much as possible. Prognosis assessment is a challenging undertaking for clinicians due to the complexity, diversity, and individuality of SCI.

Currently, clinical tools for SCI prognosis assessment largely rely on Abbreviated Injury Scale (AIS) scores [4] and Frankel grades [5], which involve evaluating the sensory and motor functions of patients after injury to roughly determine the nature of SCI and the patient’s prognosis. However, the assessment process requires high patient cooperation, and the results can be easily influenced by factors, such as the timing of neurological examinations (eg, spinal cord concussion period), the patient’s condition (eg, intoxication, sedation, and accompanying brain injury), and the subjectivity of the evaluator [6]. Machine learning (ML) is a significant area of artificial intelligence that has seen extensive application in clinical care in recent years due to the field’s rapid progress. Park et al [7] used ML combined with low-dose CT to predict prognostic biomarkers and molecular subtypes associated with invasive breast cancer. Gupta et al [8] used ML combined with magnetic resonance imaging to detect and classify brain tumors and their stages. Jumper et al [9] used ML to construct AlphaFold for predicting protein structures. ML has demonstrated high accuracy and predictive ability [10], establishing more reliable predictive models through continuous integration and analysis of large amounts of complex, nonlinear data to assist clinical decision-making.

ML has attracted considerable attention from researchers regarding SCI prognosis. Nevertheless, there is a dearth of solid data supporting the efficacy of ML models in the prognosis of SCI, and there are no systematic reviews contrasting the variations and importance of various models and prognostic markers. Therefore, this study aims to fill this gap. We conducted a systematic review and meta-analysis to evaluate the performance and quality of ML models in predicting SCI prognosis.

## Methods

The systematic review was conducted following the Preferred Reporting Items for Systematic Reviews and Meta-Analyses for Diagnostic Test Accuracy (PRISMA-DTA) guidelines. The study protocol has been registered in the PROSPERO and was approved before the start of the study (ID: 42023481977).

### Search Strategy

We conducted a literature search for studies on ML-based SCI prognosis prediction published up to February 20, 2024. A comprehensive search was performed across the following 9 electronic databases: PubMed, Web of Science, Embase, PROSPERO, Scopus, China National Knowledge Infrastructure, Cochrane Library, China Biomedical Literature Service System, and Wanfang Data.

### Inclusion and Exclusion Criteria

Inclusion criteria were (1) study subjects are patients with SCI, regardless of the nature, degree, level, or time of SCI; (2) the research method involves ML, not limited to any specific algorithm or model; (3) the purpose of the study is to predict the prognosis of patients with SCI, including spinal cord function prognosis, complication occurrence, quality of life, etc; (4) the study provides performance metrics of the model, such as accuracy, sensitivity, specificity, area under the receiver operating characteristic curve, etc; and (5) published in Chinese or English. Exclusion criteria of the study were (1) concurrent cranial brain injury or injuries to other parts of the body outside the spinal column; (2) reviews, case reports, conference abstracts, expert opinions, or other non-original research; (3) studies that do not build ML models or do not provide relevant performance metrics for ML; and (4) articles for which full text or original data cannot be obtained, repeated publication, or data duplication.

### Data Extraction and Analysis

The literature retrieved from each database was imported into EndNote X9 (Clarivate) reference management software. Two researchers trained in the systematic review process performed the screening and data extraction, with cross-checking. Initially, articles were screened based on titles and abstracts, and then full texts were reviewed according to the inclusion and exclusion criteria. For information that was uncertain but crucial to the study, the original authors were contacted via email or phone. Following the Critical Appraisal and Data Extraction for Systematic Reviews of Prediction Modelling Studies (CHARMS) guidelines for extracting data from predictive model studies [11], the following data were independently extracted from each study: study design, characteristics of the SCI population, types of ML models used, study outcomes, and predictive performance of various models.

### Risk of Bias Assessment 

Two researchers used the risk of bias (ROB) assessment tool for predictive models (Prediction Model Risk of Bias Assessment Tool [PROBAST]) to evaluate the ROB of each model in every study and the applicability to the issues we reviewed [12]. Any disagreements were resolved by a third investigator. Each model was assessed in 4 domains (participants, predictors, outcomes, and analysis) as having “high-risk,” “unclear,” or “low-risk” based on a series of specific questions. The same scale was used to evaluate the applicability of each model to the issues we reviewed in 3 domains (participants, predictors, and outcomes).

### Statistical Analysis

We used R software (version 4.2.3; R Core Team) to perform the meta-analysis. Based on the types and distributions of ML performance indicators, we selected appropriate effect metrics and models, such as 95% CI, and conducted a summary analysis using either a fixed-effects model or a random-effects model.

## Results

### Literature Selection Process

**Figure 1.**
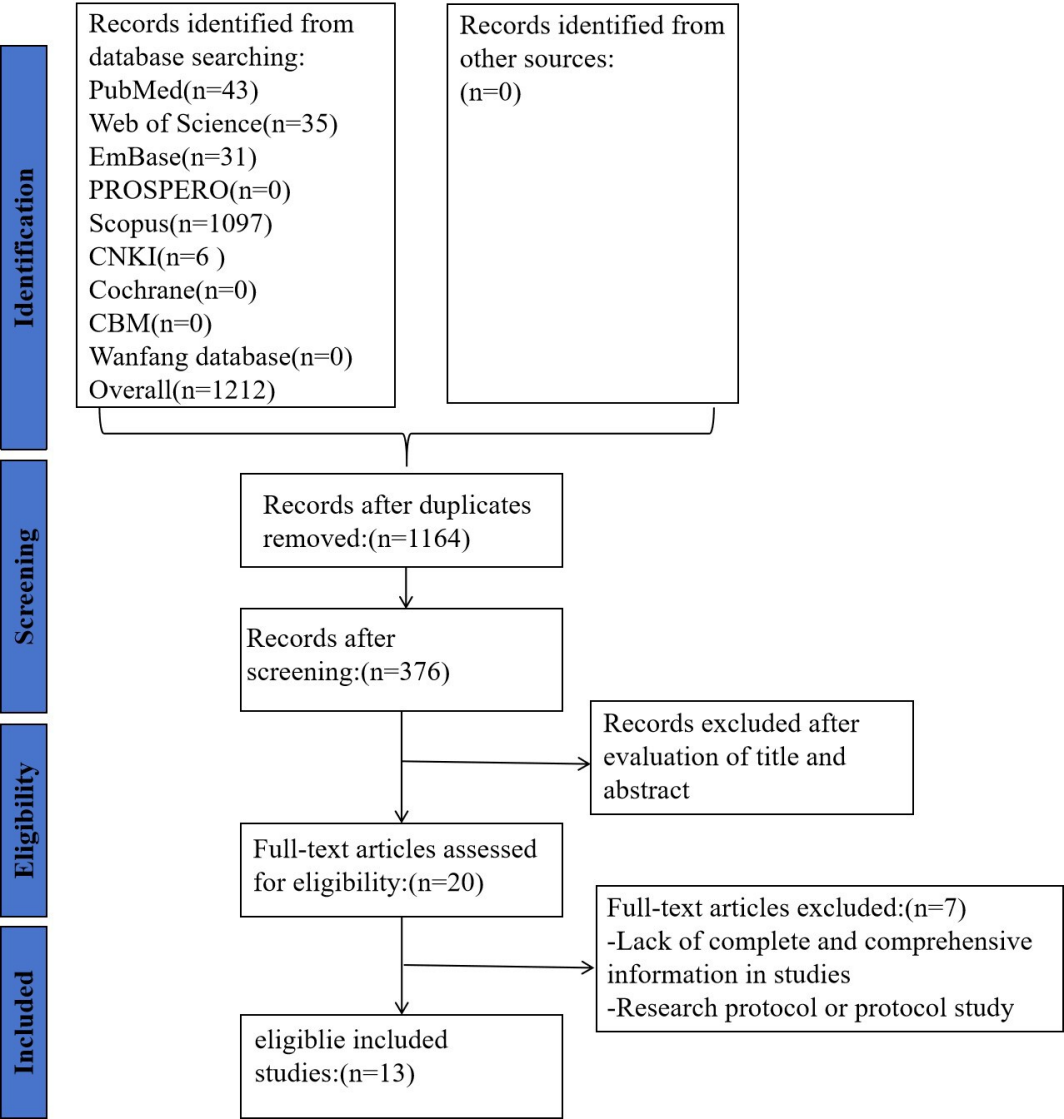
PRISMA (Preferred Reporting Items for Systematic Reviews and Meta-Analyses) flow diagram for the research search process.

The research selection process was shown in Figure 1. A total of 1212 unique records were identified, and after excluding studies without full text, conference abstracts, registration protocols, studies that failed to provide accuracy metrics for ML, and those that did not develop new ML models, 13 studies [13-25] were finally included.

### Basic Characteristics of Included Studies

The 13 included studies were from Japan (6/13, 46.15%), the United States (2/13, 14.38%), India (1/13, 7.69%), Canada (2/13, 14.38%), South Korea (1/13, 7.69%), and China (1/13, 7.69%). The study types included retrospective cohort studies [13-15,17-24] (9/13) and prospective cohort studies (2/13) [15,25]. Most articles were published in 2023 (7/13) [13,15,16,18,21-23], followed by 2022 (2/13) [19,24], 2024 (1/13) [20], 2020 (1/13) [17], 2019 (1/13) [25], and 2016 (1/13) [14]. A total of 51 ML models were, with 31 models for predicting spinal cord function prognosis, 15 models for predicting postoperative complications, 1 model for predicting independent living ability, and 4 models for assessing walking ability. The study characteristics and performance metrics are presented in Table 1.

**Table 1.** Basic characteristics of included studies.

Study	Year	Area	Sample size	Design	Artificial intelligence model	Performance evaluation
Spinal cord function prognosis						
Maki et al [13]	2023	Japan	3122	Retrospective study	CatBoost, Gradient Boosting, RF^a^, Extra Trees, LR^b^, Ada Boost, Linear Discriminant Analysis, Light Gradient Boosting Machine, Extreme Gradient Boosting, Quadratic Discriminant Analysis, Naïve Bayes, K Neighbors, DT^c^	Accuracy, AUC^d^, recall, precision, *F*_1_-score
Belliveau et al [14]	2016	US	3142	Retrospective study	ANN^e^, LR	AUC, NLR^f^, PLR^g^
Kalyani et al [15]	2023	India	165	Retrospective study	XGBoost^h^, LR, DT	AUC, Accuracy
Facchinello et al [16]	2023	Canada	172	Prospective study	CART^i^	NA^j^
Inoue et al [17]	2020	Japan	165	Retrospective study	XGBoost, LR, DT	AUC, Accuracy
Shimizu et al [18]	2023	Japan	135	Retrospective study	Light GBM, XGBoost, CatBoost	Accuracy, AUC, recall, precision, *F*_1_-score
Okimatsu et al [19]	2022	Japan	215	Retrospective study	RF	Sensitivity, Specificity, Accuracy, *F*_1_-score
Kato et al [20]	2024	Japan	210	Retrospective study	RF, SVM^m^, NN^s^, XGBoost	None
Postoperative complications						
Li et al [21]	2023	China	870	Retrospective study	RF, XGBoost, GBM^k^, NBC^l^, DT	Sensitivity, Specificity, Accuracy, AUC
Luther et al [22]	2023	US	4709	Retrospective study	the gradient boosting, LR, the adaptive LASSO^t^ model	AUC
Kim et al [23]	2023	Korea	623	Retrospective study	GNN-GCN^p^^,^^q^ DNN^o^, SVM-linear, SVM_RBF^r^, KNN^n^, RF, LR	Sensitivity, Specificity, Accuracy, AUC, *F*_1_-score
Independent living ability						
Hori et al [24]	2022	Japan	1404	Retrospective study	CART	Sensitivity, Specificity, Accuracy, AUC, *F*_1_-score, the positive predictive value
Walking ability						
DeVries et al [25]	2019	Canada	862	Prospective study	LR, VM^u^, Hicks, Unsupervised MLA^v^	AUC, *F*_1_-score

a RF: random forest.

b LR: logistic regression.

c DT: decision tree.

d AUC: area under the curve.

e ANN: artificial neural network.

f NLR: neutrophil-to-lymphocyte ratio.

g PLR: platelet-to-lymphocyte ratio.

h XGBoost: extreme gradient boosting.

i CART: classification and regression tree.

j NA: ____.

k GBM: gradient boosting machine.

l NBC: naïve Bayes classifiers.

m SVM: support vector machine.

n KNN: K nearest neighbors.

o DNN: deep neural networks.

p GNN: graph neural networks.

q GCN: graph convolutional network.

r SVM_RBF: support vector machine using a radial basis function.

s NN: neural network.

t LASSO: least absolute shrinkage and selection operator.

u VM: vector machine.

v MLA: machine learning algorithm.

### Evaluation Performance of Different Prediction Models

**Figure 2.**
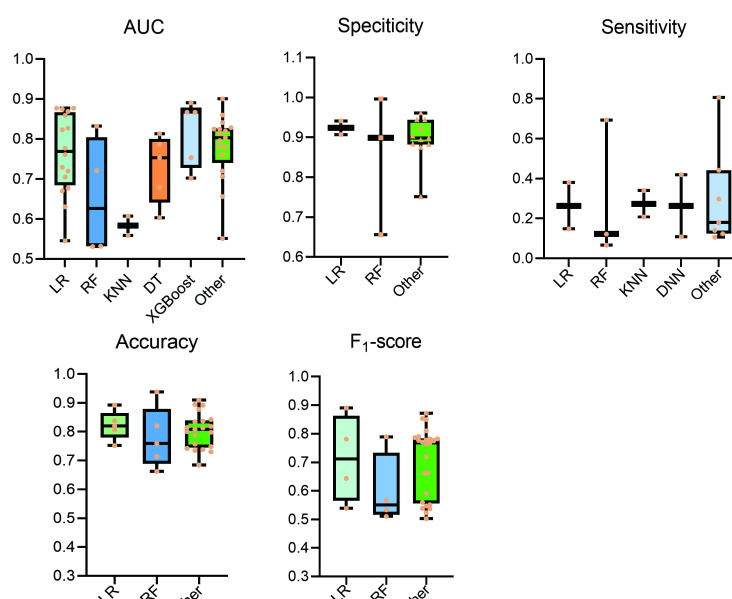
Summary of performance for different machine learning models. AUC: area under the curve; DNN: deep neural networks; DT: decision tree; KNN: K nearest neighbors; LR: logistic regression; RF: random forest; XGBoost: extreme gradient boosting.

The area under the curve (AUC) has long been used to evaluate model performance and is therefore a primary performance indicator. The AUC range for ML prediction models is 0.532‐0.904 (Figure 2). Additionally, other performance metrics of ML prediction models, such as specificity, sensitivity, accuracy, and *F*_1_-score, were also extracted.

### Results of Bias Risk Assessment

**Figure 3.**
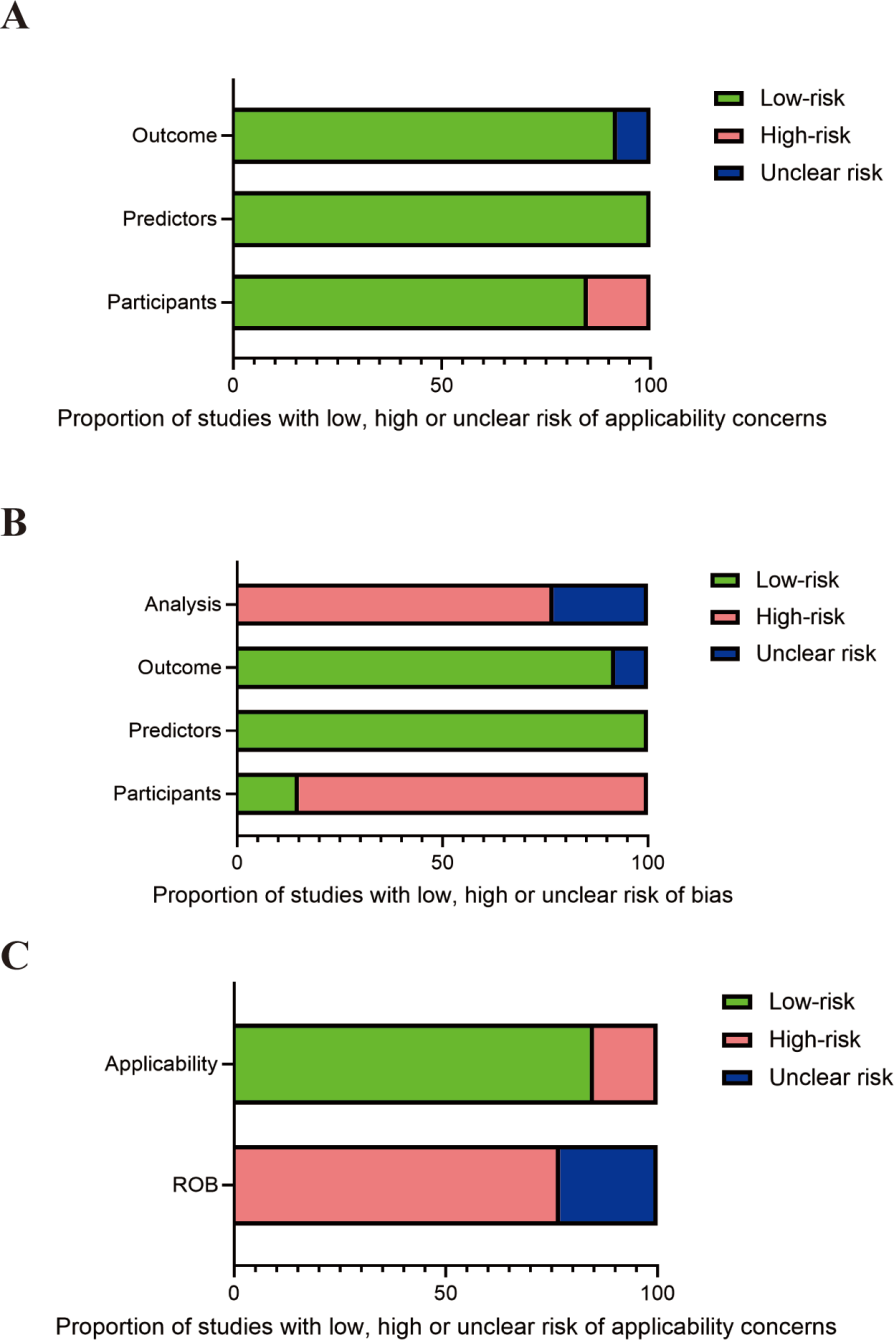
Schematic diagram of bias risk assessment components. (A) Proportion of studies with low, high, or unclear risk of bias; (B) proportion of studies with low, high, or unclear risk of applicability concerns; (C) proportion of studies with low, high, or unclear risk of applicability concerns. ROB: risk of bias.

The ROB in ML prediction based on medical information was assessed using PROBAST (Table 2 and Figure 3). According to the PROBAST evaluation, only 2 studies [16,25] were designed as prospective cohort studies and were thus rated as low-ROB. In contrast, the majority of studies [13-15,17-24] were retrospective cohort studies and were consequently rated as high-risk. In the domains of predictors and outcomes, item 3.2 refers to whether the outcome was prespecified or defined using standard criteria. One study [15] lacked sufficient information on this item and was therefore rated as having an “unclear” ROB. Additionally, 2 studies [16,20] failed to appropriately evaluate model performance; specifically, item 4.7, which assesses whether performance measures were adequately reported and validated, was rated as “high-risk” for these studies. Given that most of the included studies were retrospective in nature, with only a few being prospective, the overall ROB in outcome assessment was considered to be high or unclear across the reviewed literature.

**Table 2.** Risk-of-bias assessment in machine learning prediction using Prediction Model Risk of Bias Assessment.

Study	Risk of bias				Applicability concerns			Overall	
	Participants	Predictors	Outcomes	Analysis	Participants	Predictors	Outcomes	ROB^d^	Applicability
Maki et al [13]	–^a^	+^b^	+	–	+	+	+	–	+
Belliveau et al [14]	–	+	+	–	+	+	+	–	+
Kalyani et al [15]	–	+	?^c^	–	–	+	?	–	–
Facchinello et al [16]	+	+	+	?	+	+	+	?	+
Inoue et al [17]	–	+	+	–	+	+	+	–	+
Shimizu et al [18]	–	+	+	–	+	+	+	–	+
Okimatsu et al [19]	–	+	+	–	+	+	+	–	+
Luther et al [22]	–	+	+	–	–	+	+	–	–
Li et al [21]	–	+	+	–	+	+	+	–	+
Kato et al [20]	–	+	+	–	+	+	+	–	+
Kim et al [23]	–	+	+	?	+	+	+	?	+
Hori et al [24]	–	+	+	?	+	+	+	?	+
DeVries et al [25]	+	+	+	–	+	+	+	–	+

a **−**: high-risk.

b +: low-risk.

c ?: unclear risk.

d ROB: risk of bias.

### Meta-Analysis Results

We conducted separate meta-analyses on different prognostic indicators. Overall, ML applications in SCI prognosis showed a high AUC. Considering that many studies lacked assessments of accuracy, sensitivity, and specificity, and that there were certain differences between algorithms and models, this reflects the complexity and significance of SCI disease prognosis prediction. Therefore, we only focused on and explored the AUC. The specific analysis results are listed below.

#### Spinal Cord Functional Prognosis

**Figure 4.**
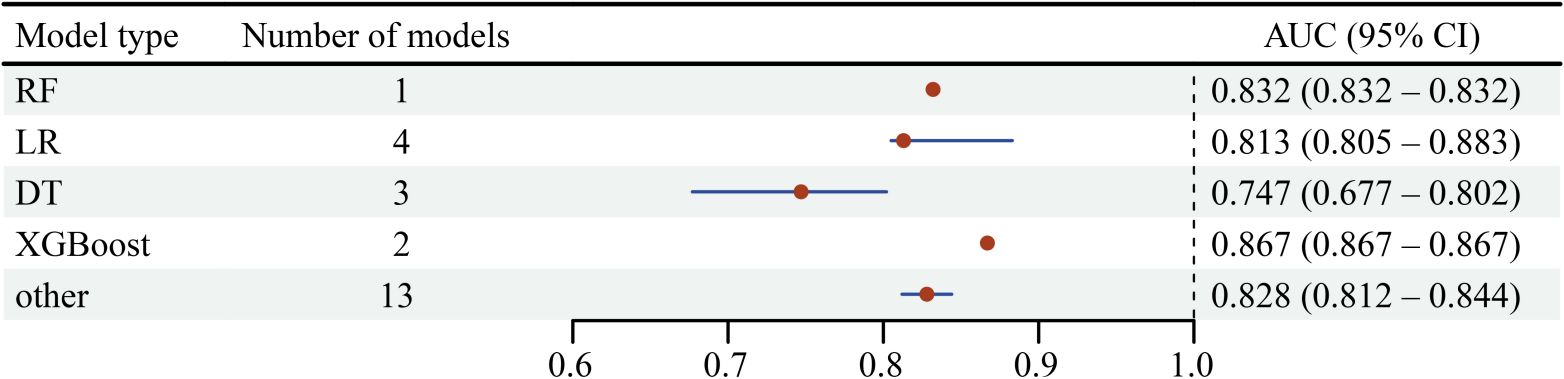
Forest plot of models related to spinal cord functional prognosis. DT: decision tree; LR: logistic regression; RF: random forest; XGBoost: extreme gradient boosting.

We included a total of 8 studies [13-20] with 7326 patients, using 17 ML algorithms to predict functional recovery indicators, such as American Spinal Injury Association grading, motor scores, and sensory scores. Three studies [13,19,20] used the random forest (RF) algorithm, 4 studies [13-15,17] used the logistic regression (LR) algorithm, and 3 studies [13,15,17] used the decision tree (DT) algorithm. The sample size for studies using the RF algorithm was 3547, for the LR algorithm was 6594, and for the DT algorithm was 3452. The AUC for the RF algorithm was 0.832, for the LR algorithm was 0.813 (95% CI 0.805-0.883), for the DT algorithm was 0.747 (95% CI 0.677-0.802), and for the extreme gradient boosting (XGBoost) algorithm was 0.867 (Figure 4).

#### Postoperative Complications

**Figure 5.**
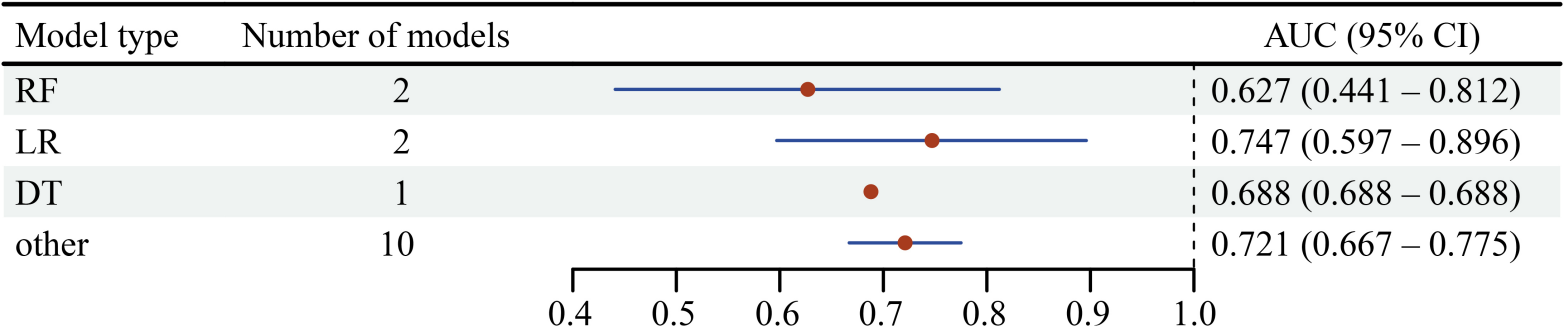
Forest plot of models related to postoperative complications. RF: random forest; LR: logistic regression; DT: decision tree.

We included 3 studies with a total of 6202 patients, using 12 ML algorithms. Two studies used RF and LR algorithms, with the RF algorithm having a sample size of 1493 and the LR algorithm having a sample size of 5332. The AUC for the RF algorithm was 0.627 (95% CI 0.441-0.812), for the LR algorithm was 0.747 (95% CI 0.597-0.896), and for the DT algorithm was 0.688 (Figure 5). 

#### Independent Living Ability

A study [24] was conducted to assess the ability of patients with SCI to live independently. A total of 1404 patients were included, and the classification and regression tree (CART) algorithm was used. The model’s predictive variables included age, sex, Functional Independence Measure score, American Spinal Injury Association score, and the presence or absence of complications, such as diabetes and hypertension. The AUC of the CART model was 0.813.

#### Walking Ability

Currently, only 1 study [25] has been collected to evaluate the independent walking ability of patients with SCI at discharge or at a 1-year follow-up. This study included 862 patients and used LR, vector machine (VM), and Hicks algorithms for prediction, with AUCs of 0.720, 0.780, and 0.760, respectively. The model based on the VM algorithm showed the highest effectiveness.

## Discussion

### Principal Findings

SCI remains one of the leading causes of death and disability worldwide [26]. The heterogeneity factors, such as variable disease course, high inconsistency, and complex pathophysiological processes are major reasons for poor prognosis [27]. Clinically, a key concern for patients and their families is the prognosis, which is often assessed using imaging techniques, such as head computed tomography and magnetic resonance imaging, requiring considerable experience from clinicians. If clinicians had an objective assessment tool that could quickly and accurately predict prognosis based on the patient’s condition, it would provide greater confidence in prognosis judgments [28]. Therefore, the idea of using ML to facilitate objective and individualized prognosis predictions for patients with SCI has emerged. In recent years, the application of ML in SCI prognosis prediction has made continuous breakthroughs, and the number and quality of related articles have been increasing over time. To our knowledge, this is the first meta-analysis evaluating the performance of ML models in predicting SCI prognosis. We conducted a comprehensive literature search and systematically evaluated the performance of ML models in SCI prognosis. Ultimately, ML algorithms were used to predict SCI prognosis in 13 included studies [13-25]. Among various model algorithms, although LR is a traditional algorithm, it is still commonly used in SCI prognosis prediction [13-15,17,22,23,25]. Other major algorithms include RF, DT, XGBoost, ANN, etc. A total of 18 algorithms were used to predict functional recovery after SCI, 12 algorithms for predicting complications after SCI, 1 algorithm for predicting independent living ability after SCI, and 3 algorithms for predicting walking ability after SCI. These models demonstrated varying levels of AUC. AUC indicates the algorithm’s ability to balance sensitivity and specificity to minimize false negatives and false positives [29]. Therefore, its evaluation is particularly important in SCI prognosis assessment models. For predicting functional recovery, the ANN algorithm showed the highest AUC of 0.902. For predicting complications, the support vector machine-linear algorithm showed the highest AUC of 0.904. For predicting post-injury walking ability, both LR and VM models demonstrated the highest AUC of 0.870. Our study also found that over 90% of the included studies were published in the past 3 years, indicating growing interest in using ML-related predictive models to guide clinical decision-making.

It is worth noting that although most of the data in this study come from hospitals, the participant populations are from different countries and ethnic groups, with a focus on Asian and European populations, and there is little research on African populations. Published studies have not yet used predictive models to examine different regional and ethnic groups. Therefore, further model construction and validation using data from different populations are needed to increase confidence. Research indicates that ML models are significantly superior to traditional statistical models in handling large sample data [30,31]. However, most of the 13 studies [13-25] included had sample sizes of fewer than 1000 cases, which suggests that future research should aim to collect sufficiently large and comprehensive sample sizes to reduce bias. Most studies opted to randomly split single-center data into training and testing sets or used different time periods for internal validation. However, the lack of external validation poses significant challenges to the model’s applicability, potentially leading to overestimation of the model’s effectiveness. Thus, SCI-related prognostic models require rigorous external validation to enhance their credibility.

Our study also indicates that most of the included studies use functional recovery as the outcome measure for SCI prognosis [13-20], and over time, research using ML has progressively reached higher quality standards. This has aroused considerable curiosity among SCI researchers and reflects the growing progress and recognition of ML applications in the field of SCI. In clinical practice, the AIS score remains of significant importance for evaluating the prognosis of patients with SCI. The AIS score uses clear criteria to assess patients’ neurological function, offering objectivity and reproducibility and is applicable to various types of SCI, including traumatic and nontraumatic [32]. Despite its practicality, the AIS score has limitations. First, it mainly focuses on physiological aspects, such as sensory and motor functions, while neglecting the impact of psychological and social factors, which may affect clinicians’ prognosis assessments. Second, it may lack sensitivity and might not assess mild SCI, thereby affecting long-term prognosis. Additionally, the AIS score is not suitable for SCI in children younger than 4 years [33].

In contrast, ML continues to evolve, and the improvement of various SCI databases highlights the increasing potential of ML in predicting SCI prognosis. ML can handle large amounts of complex data and extract nonlinear relationships, offering promising solutions to unknown challenges [34]. Our study found that 5 studies [13-15,17,24] used public databases to build predictive models and achieved good results in model performance. Additionally, many studies not only used traditional models, such as RF and DT, but also used recently popular models such as XGBoost. For instance, the XGBoost model constructed by Inoue et al [17] achieved an AUC of 0.867, outperforming models, such as LR and RF, and holds promise for future clinical guidance. Traditional clinical prognostic tools often face subjective biases of researchers and limited variables, while ML incorporates as many variables as possible, including demographic data, imaging, and laboratory results. This broad coverage allows for accurate and detailed individualized predictions based on each patient’s specific situation, greatly reducing ambiguity and generalization. Furthermore, ML models continuously learn and optimize to improve prediction accuracy and explore potential patterns in data, potentially matching or even exceeding human brain capabilities [35].

### Limitations

This systematic review and meta-analysis have several limitations. First, the data quality from retrospective studies is lower compared to prospective studies. Among the 13 studies [13-25] included, only 2 were prospective, while the rest were retrospective, resulting in overall lower quality of the included literature. According to PROBAST guidelines, future research should aim to use prospective studies to obtain data, ensuring reliable model performance. Second, we focused solely on AUC to evaluate the performance of predictive models, without a comprehensive assessment using sensitivity, specificity, and *F*_1_-score metrics. This is due to incomplete data in the selected literature, so the meta-analysis results may be somewhat one-sided. Moreover, because most studies did not consistently report key metrics, such as sensitivity and specificity, we did not perform a formal heterogeneity analysis. This limitation may affect the generalizability of the pooled AUC results across different study settings. Our study also did not focus much on the specific segments of the spinal cord damaged in patients with SCI or perform subgroup analysis based on different injury segments. Studies have shown that the mortality rate for patients with cervical SCI in the acute phase ranges from 11.2% to 25% [36], with the risk of death being about 7 times higher in upper cervical SCI compared to lower cervical SCI [37]. This indicates that prognosis varies with different spinal cord segments, suggesting a need for future research to focus on this aspect.

We must also recognize that ML research is inherently susceptible to multiple sources of bias that extend beyond the scope of conventional study design. The included studies used a wide range of algorithms, from traditional LR models to more complex methods, such as XGBoost and neural networks, resulting in significant heterogeneity in both model development and performance evaluation. Moreover, bias may arise from imbalanced data distributions, insufficient feature representation, and lack of transparency in dataset construction. These issues are particularly concerning given that most studies did not undergo external validation, which further increases the risk of model overfitting and undermines the reliability of their clinical applicability. Additionally, model interpretability is an important factor to consider. To date, no ML model has been truly used for predicting SCI patient prognosis in clinical practice. Higher interpretability of a model allows clinicians to better understand its predictive value and make clinical decisions that benefit patients [38]. Lundberg et al [39] used the Shapley additive explanations algorithm to interpret any ML model’s outputs and quantify each variable’s contribution using Shapley additive explanations values, thereby promoting clinical application. Future research is expected to continue addressing this challenging issue of model interpretability [40].

### Conclusions

Increasing research efforts are devoted to developing SCI prognosis prediction models, which can provide personalized prognosis assessments for patients and ultimately alleviate their burden. However, the application of ML in this field is still in its early stages. Existing studies lack some data and details on predictive model reporting, and the majority are retrospective, leading to limited practical application of these models. Although clinical translation of ML is still a long way off, this study demonstrates that ML under artificial intelligence is poised to bring about a significant revolution in the field of SCI.

## Supplementary material

10.2196/66233 Checklist 1 PRISMA checklist.

## Abbreviations

AIS: Abbreviated Injury Scale
AUC: area under the curve
CART: classification and regression tree
CHARMS: Critical Appraisal and Data Extraction for Systematic Reviews of Prediction Modelling Studies
DT: decision tree
LR: logistic regression
ML: machine learning
PRISMA-DTA: Preferred Reporting Items for Systematic Reviews and Meta-Analyses for Diagnostic Test Accuracy
PROBAST: Prediction Model Risk of Bias Assessment Tool
RF: random forest
ROB: risk of bias
SCI: spinal cord injury
VM: vector machine
XGBoost: extreme gradient boosting
