# Supplementary material for: Predicting Spinal Cord Injury Prognosis Using Machine Learning: Systematic Review and Meta-Analysis
Source: JMIR AI. 2025 Dec 5;4:e66233. doi: 10.2196/66233 (PMC12680090; doi:10.2196/66233)
Supplement: Checklist 1 [file ai-v4-e66233-s001.docx]

| **Prisma checklist** | | | |
| --- | --- | --- | --- |
| **Item** | **Description** | **Reported** | **Location/Section** |
| **TITLE** |  |  |  |
| Title | Identify the report as a systematic review. | Yes | Title: "Predicting Spinal Cord Injury Prognosis Using Machine Learning: ASystematic Review and Meta-Analysis" |
| **ABSTRACT** |  |  |  |
| Abstract | See the PRISMA 2020 for Abstracts checklist. | Yes | Abstract section: Provides background, methods, results, and conclusion, including key metrics like AUC (0.747, 0.813) assessment. |
| **INTRODUCTION** |  |  |  |
| Rationale | Describe the rationale for the review in the context of existing knowledge. | Yes | Introduction: Discusses the application of machine learning in spinal cord injury and the necessity of integrating research to guide clinical applications.. |
| Objectives | Provide an explicit statement of the objective(s) or question(s) the review addresses. | Yes | Introduction: "We conducted a systematic review and meta-analysis to evaluate the performance and quality of ML models in predicting SCI prognosis." |
| **METHODS** |  |  |  |
| Eligibility criteria | Specify the inclusion and exclusion criteria for the review and how studies were grouped for the syntheses. | Yes | Methods - Eligibility Criteria: Lists inclusion (population/outcome, study design/language, definition requirement, model type, performance reporting, imaging modality) and exclusion criteria (language/subject, model/focus, study type, duplications). |
| Information sources | Specify all databases, registers, websites, organisations, reference lists and other sources searched or consulted to identify studies. Specify the date when each source was last searched or consulted. | Yes | Methods - Search Strategy: PubMed, Web of Science, Embase, PROSPERO, Scopus, CNKI, Cochrane Library, CBM, and Wanfang Data results. Searched from inception to February 20, 2024. Reference lists hand-searched. |
| Search strategy | Present the full search strategies for all databases, registers and websites, including any filters and limits used. | Yes | Methods - Search Strategy: Keywords/MeSH terms like “machine learning,” “artificial intelligence,” “spinal cord injury,” “prognosis,” Full strategy in Supplementary Table 1. |
| Selection process | Specify the methods used to decide whether a study met the inclusion criteria of the review, including how many reviewers screened each record and each report retrieved, whether they worked independently, and if applicable, details of automation tools used in the process. | Yes | Methods - Data Extraction and Analysis Selection: Two researchers trained in the systematic review process performed the screening and data extraction, with cross-checking. Blinded initially; discrepancies resolved by consensus or third reviewer. Reference lists hand-searched. |
| Data collection process | Specify the methods used to collect data from reports, including how many reviewers collected data from each report, whether they worked independently, any processes for obtaining or confirming data from study investigators, and if applicable, details of automation tools used in the process. | Yes | Methods - Data Extraction: Two reviewers independently extracted data using standardized form. Discrepancies resolved by discussion. No contact with authors for missing data. |
| Data items | 10a: List and define all outcomes for which data were sought. Specify whether all results that were compatible with each outcome domain in each study were sought... | Yes | Methods - Data Extraction: Performance metrics (accuracy, sensitivity, specificity, precision, AUC) and raw counts (TP, TN, FP, FN). All compatible results sought; best-performing model selected per study. |
|  | 10b: List and define all other variables for which data were sought (e.g. participant and intervention characteristics, funding sources). Describe any assumptions made about any missing or unclear information. | Yes | Methods - Data Extraction: study design, characteristics of the SCI population, types of machine learningML models used, study outcomes, and predictive performance of various models. Metrics calculated/imputed from raw data if missing; no assumptions detailed beyond that. |
| Study risk of bias assessment | Specify the methods used to assess risk of bias in the included studies, including details of the tool(s) used... | Yes | Methods - Risk of Bias Assessment: PROBAST tool used by two reviewers independently; disagreements resolved by consensus/senior reviewer. Rated low/moderate/high. |
| Effect measures | Specify for each outcome the effect measure(s) (e.g. risk ratio, mean difference) used in the synthesis or presentation of results. | Yes | Methods - Statistical Analysis: Pooled sensitivity, specificity, accuracy, precision, AUC. |
| Synthesis methods | 13a: Describe the processes used to decide which studies were eligible for each synthesis... | Yes | Methods - Statistical Analysis: by Inclusion and Exclusion Criteria. |
|  | 13b: Describe any methods required to prepare the data for presentation or synthesis... | No | Methods - Statistical Analysis: N/A. |
|  | 13c: Describe any methods used to tabulate or visually display results... | Yes | Results: Tables 1 for characteristics/performance; Figures 1-5 (PRISMA diagram, forest plots). |
|  | 13d: Describe any methods used to synthesize results... | Yes | Methods - Statistical Analysis: Based on the types and distributions of machine learningML performance indicators, we selected appropriate effect metrics and models, such as 95% CI, and conducted a summary analysis using either a fixed-effects model or a random-effects model.. |
|  | 13e: Describe any methods used to explore possible causes of heterogeneity... | No | N/A |
|  | 13f: Describe any sensitivity analyses conducted... | No | N/A |
| Reporting bias assessment | Describe any methods used to assess risk of bias due to missing results... | No | N/A |
| Certainty assessment | Describe any methods used to assess certainty (or confidence) in the body of evidence... | Yes | Methods - Quality Assessment: The Risk of Bias (ROB) for individual studies was assessed using the PROBAST tool (Prediction model Risk Of Bias Assessment Tool). Studies with high risk of bias were considered to contribute less reliable evidence, while studies with low or unclear bias risk were deemed to provide higher-quality evidence. The majority of the included studies had a high risk of bias, mainly due to their retrospective design and lack of external validation.. |
| **RESULTS** |  |  |  |
| Study selection | 16a: Describe the results of the search and selection process... | Yes | Results - Study Selection: The research selection process was shown in Figure 1. A total of 1,25406 unique records were identified, and after excluding studies without full text, conference abstracts, registration protocols, studies that failed to provide accuracy metrics for ML, and those that did not develop new ML models, 13 studies were finally included. PRISMA diagram (Figure 1). |
|  | 16b: Cite studies that might appear to meet the inclusion criteria, but which were excluded... | Yes | Results - Study Selection: 7 excluded with reasons (e.g., lack of complete and comprehensive information in studies n=7). |
| Study characteristics | Cite each included study and present its characteristics. | Yes | Results - Study Characteristics: Table 1 lists all 13 studies with details (author/year, type, country, validation, etc.). |
| Risk of bias in studies | Present assessments of risk of bias for each included study. | Yes | Results - Quality Assessment: via PROBAST; details in Figure 3. |
| Results of individual studies | For all outcomes, present, for each study: (a) summary statistics... (b) effect estimate... | No | Results - Pooled Diagnostic Performance: Table 1 presents metrics (accuracy, sensitivity, etc.) for each study/model. |
| Results of syntheses | 20a: For each synthesis, briefly summarise the characteristics and risk of bias... | Yes | Results - Pooled Diagnostic Performance: 13 studies synthesized; characteristics in Table 1; ROB summarized in Quality Assessment. |
|  | 20b: Present results of all statistical syntheses... | Yes | Results: LR algorithm was 0.813 (95% CI: 0.805, -0.883), for the DT algorithm was 0.747 (95% CI: 0.677, -0.802); Figures 4-5. |
|  | 20c: Present results of all investigations of possible causes... | Yes | Results - Meta-Regression: Sample size and model architecture significant; Supplementary Table 1. |
|  | 20d: Present results of all sensitivity analyses... | No | N/A |
| Reporting biases | Present assessments of risk of bias due to missing results... | No | N/A |
| Certainty of evidence | Present assessments of certainty (or confidence)... | Yes | Results - GRADE Assessment: Moderate certainty due to inconsistency. |
| **DISCUSSION** |  |  |  |
| Discussion | 23a: Provide a general interpretation of the results... | Yes | Discussion: ML shows promising accuracy ; comparisons to MRI, other models, conventional methods. |
|  | 23b: Discuss any limitations of the evidence included... | Yes | Limitations: Heterogeneity, geographic bias, dataset overlap, varying criteria. |
|  | 23c: Discuss any limitations of the review processes... | Yes | Limitations: Substantial heterogeneity limits pooled estimates; possible dataset overlap; lack of direct comparisons. |
|  | 23d: Discuss implications of the results... | Yes | Discussion - Clinical applicability: Utility in automation; Limitations/Future directions: Need for interpretability, multimodal data, validation; implications for practice and research. |
| **OTHER INFORMATION** |  |  |  |
| Registration and protocol | 24a: Provide registration information... | Yes | Methods - Study Design: Registered on PROSPERO (ID: 42023481977). |
|  | 24b: Indicate where the review protocol can be accessed... | Yes | Methods - Study Design: Protocol on PROSPERO (http://www.crd.york.ac.uk/prospero/). |
|  | 24c: Describe and explain any amendments... | No | Not reported; no amendments mentioned. |
| Support | Describe sources of financial or non-financial support... | Yes | Declarations - Funding: None. |
| Competing interests | Declare any competing interests... | Yes | Declarations - Competing interests: None declared. |
| Availability of data, code and other materials | Report which of the following are publicly available... | Yes | Declarations - Availability of data: All data included in article and supplementary files. |
